# Proteomic analysis of antimicrobial effects of pegylated silver coated carbon nanotubes in *Salmonella enterica* serovar Typhimurium

**DOI:** 10.1186/s12951-018-0355-0

**Published:** 2018-03-27

**Authors:** Seong B. Park, Christy S. Steadman, Atul A. Chaudhari, Shreekumar R. Pillai, Shree R. Singh, Peter L. Ryan, Scott T. Willard, Jean M. Feugang

**Affiliations:** 1 Department of Animal and Dairy Sciences, Mississippi State University, Mississippi State, MS 39762 USA; 2 Center for Nanobiotechnology Research, Alabama State University, Montgomery, AL USA; 3 Department of Pathobiology and Population Medicine, Mississippi State University, Mississippi State, MS 39762 USA; 4 Department of Biochemistry, Molecular Biology, Entomology and Plant Pathology, Mississippi State University, Mississippi State, MS 39762 USA

**Keywords:** Foodborne pathogens, Pegylated silver-coated carbon nanotubes, Bacterial growth kinetics, Bioluminescence imaging, Chicken embryo development, Proteomics

## Abstract

**Background:**

Synthesis of silver nano-compounds with enhanced antimicrobial effects is of great interest for the development of new antibacterial agents. Previous studies have reported the antibacterial properties of pegylated silver-coated carbon nanotubes (pSWCNT-Ag) showing less toxicity in human cell lines. However, the mechanism underlining the pSWCNT-Ag as a bactericidal agent remained unfolded. Here we assessed the pSWCNT-Ag effects against foodborne pathogenic bacteria growth and proteome profile changes.

**Results:**

Measurements of bioluminescent imaging, optical density, and bacteria colony forming units revealed dose-dependent and stronger bactericidal activity of pSWCNT-Ag than their non-pegylated counterparts (SWCNT-Ag). In *ovo* administration of pSWCNT-Ag or phosphate-buffered saline resulted in comparable chicken embryo development and growth. The proteomic analysis, using two-dimensional electrophoresis combined with matrix assisted laser desorption/ionization time of flight/time of flight mass spectrometry, was performed on control and surviving *Salmonella enterica* serovar Typhimurium to pSWCNT-Ag. A total of 15 proteins (ten up-regulated and five down-regulated) differentially expressed proteins were identified. Functional analyses showed significant reduction of proteins associated with biofilm formation, nutrient and energy metabolism, quorum sensing and maintenance of cell structure and cell motility in surviving *S*. Typhimurium. In contrast, proteins associated with oxygen stress, DNA protection, starvation, membrane rebuilding, and alternative nutrient formation were induced as the compensatory reaction.

**Conclusions:**

This study provides further evidence of the antibacterial effects of pSWCNT-Ag nanocomposites and knowledge of their mechanism of action through various protein changes. The findings may lead to the development of more effective and safe antimicrobial agents.

## Background

The emergence of multidrug-resistant bacteria is a global issue in human and veterinary medicine. Recent epidemiological studies have indicated the high prevalence of multidrug-resistant bacteria as a consequence of the abuse utilization of antibiotics and sharing of antibiotic-resistant genes among bacteria [1]. Furthermore, increasing transportation of humans, animals, and foods across the world also contributes to the prevalence of multidrug-resistant bacteria [2, 3]. Studies reported that antibiotic-resistant bacteria could be detected in a population that has rarely or never been exposed to antimicrobial agents in the past [3–5]. Although various broad-spectrum antibiotics have been developed during last decades, bacteria still show multidrug-resistance [3, 4].

Studies demonstrated that foodborne pathogens exhibiting drug resistance against five or more antibiotics were mainly isolated from foodborne outbreaks involving ground meat, poultry and dairy products (31/55, 56%) [6]. It is believed that the overuse of antibiotics in the animal industry and biofilm formation during food processing contribute to the emergence of multidrug-resistant foodborne bacteria [3, 5, 7]. As a consequence, the consumption of food contaminated with pathogens such as *Salmonella* spp., *Escherichia coli*, and *Campylobacter* spp. causes foodborne disease outbreaks, and investigating new technologies or agents is becoming vital for efficient microbial growth control [8–10].

The recent development of nanotechnology tools and their combination with microbiology have shown promising applications in inhibition of bacterial growth [11]. Numerous studies have demonstrated the antimicrobial effects of nano-sized metals (i.e., silver, silica, and gold), metal oxides (silver oxide, titanium oxide, and copper oxide), and carbon-based compounds (i.e., fullerene, graphene oxide and single-walled carbon nanotubes or SWCNTs) against Gram-negative and -positive bacteria [12–14]. These nanoparticles are easy to synthesize and possess large surface area-to-volume ratio and high versatility that favor their uses for antimicrobial growth without affecting human eukaryote cells [11, 15]. Among the currently tested nanoparticles, silver and SWCNT have exhibited high potentials for the treatment of multidrug resistance in various bacteria, including foodborne strains [16–18].

Although the mechanism of actions of both silver (Ag) and SWCNT nanocomposites remain unclear, their direct interactions with bacterial cell membrane leading to death are believed to be the primary path for bacterial growth inhibition [13, 19]. Various studies have shown that silver compounds mainly induce ribosomal destabilization, DNA and mitochondrial damages, and free radical formation that results in cell wall destruction [11, 20–23], while SWCNTs preferentially fusion the bacterial cell membrane to destabilize its structure and perturb the metabolism process [18, 24]. A recent study combined both nanoparticles to form high-performance nanocomposites (SWCNT-Ag) for greater antimicrobial strengths, while their further polymerization with polyethylene glycol or pegylation (pSWCNT-Ag) to enhance dispersion (hydrophilicity) and reduce cytotoxicity has led to comparable or stronger antibacterial effects of pSWCNT-Ag [25]. Both nanocomposites maintained antibacterial properties and only pSWCNT-Ag exhibited non-toxicity to various human cell lines [25]. Current findings indicated the internalization of nanocomposites into foodborne pathogens, leading to cell rupture and expulsion of cytoplasmic contents while the molecular mechanisms underlying the antimicrobial property of pSWCNT-Ag remain unknown [25].

In this study, we used three major foodborne pathogens (*E. coli*, *Salmonella enterica serovar* Typhimurium and *S. enterica serovar* Anatum) to evaluate the antibacterial effects of pSWCNT-Ag. Bacteria were transformed for bioluminescence emission, followed by growth assessments through optical density (spectrophotometer) measurement, real-time bioluminescence imaging, and colony forming unit counts. The cytotoxicity of pSWCNT-Ag on a hypothetical host was determined through *in ovo* chicken embryo development. Proteomic changes induced by the pSWCNT-Ag exposure were examined on the *S.* Typhimurium strain, used as a model. The results (i) confirmed antibacterial and non-cytotoxicity effects of pSWCNT-Ag, (ii) revealed real-time bioluminescence imaging as a useful tool for early detection of bacteria, and (iii) indicated low expression levels of proteins associated with biosynthesis of amino acids and secondary metabolites, carbon metabolism and cell motility as principal targets of pSWCNT-Ag antimicrobial property.

## Methods

### Production of bioluminescent bacteria

Strains of *Escherichia coli* O157:H7 (ATCC 43888), *Salmonella enterica* serovar Typhimurium (ATCC 14028) and *Salmonella enterica* serovar Anatum (ATCC 9270) were cultured in Luria–Bertani broth (LB broth, BD, Franklin Lakes, NJ, USA) with shaking or on solid LB agar at 37 °C. Bioluminescent bacteria were constructed by electroporation of pXen5-*luxCDABE* (Caliper life sciences, Hopkinton, MA, USA) containing ampicillin resistance gene into the target strains. Colonies of successfully transformed bacteria exhibiting bioluminescence were positively selected on solid agar medium containing ampicillin (100 µg/ml). The bioluminescence imaging of bacteria colony was performed using the In Vivo Imaging System (IVIS, Lumina XRMS Series III system, Perkin Elmer, Waltham, MA, USA).

### Properties of silver nanocomposites

Silver-coated single-walled carbon nanotubes (SWCNT-Ag) and pegylated SWCNT (pSWCNT-Ag) were prepared and characterized by Chaudhari et al. [25] as previously described. The prepared pSWCNT-Ag had positive zeta potential (8.99) and more positive charges than SWCNT-Ag (− 41.9). The pSWCNT-Ag exhibited bigger size (54 nm vs. 6 nm in diameter; TEM imaging) and high hydrophilicity than the SWCNT-Ag [25]. In the present study, both nanocomposites were further characterized using the X-ray diffraction (XRD—Rigaku’s Ultima III XRD; The Woodlands, TX, USA) operated under 40 kV and 44 mA (scan range_10°–90°, step size_0.02°, and scan speed_1°/min). The JEM 2100 TEM (Jeol USA, Inc.; Peabody, MA, USA) coupled with the energy dispersive X-ray spectroscopy (EDS—Oxford Instrument’s X-Max 80T EDS detector) was also used.

### Bacterial growth analyses

*Escherichia coli*, *S.* Typhimurium and *S.* Anatum were grown in LB broth until early stationary phase. Cell concentrations were then adjusted to 1 × 10^7^ colony forming unit (CFU)/ml of fresh culture medium containing various concentrations of SWCNT-Ag or pSWCNT-Ag (0, 25, 31.25, 50 and 62.5 μg/ml). One set of each culture plate and treatment was incubated in the Cytation 5 spectrophotometer (Bioteck, Winooski, VT, USA) to monitor bacterial growth (at 600 nm OD) throughout a 48 h period. Another set of plates was placed in the IVIS chamber (at 37 °C) for culture, with the bioluminescence emission signal captured every hour, 48 times total, to monitor real-time bacterial growth kinetics. In a different set of culture plates, suspensions of bacteria were overnight-cultured in a solid phase (agar medium) to determine the number of live bacteria (CFU/ml) after 48 h cultivation under pSWCNT or SWCNT.

### Evaluation of toxicity effect of pSWCNT-Ag in developing chicken embryo

Fertilized White Leghorn eggs (n = 6, 54 ± 3.5 g) were a gift of Professor Christopher McDaniel (Department of Poultry Science, Mississippi State University). Eggs were incubated at 37 °C with 60% humidity and automatically tilted to 30° angle every hour. At day 12 post-fertilization, the viability of egg was measured by candling, followed by the disinfection of the top of eggshell with 70% ethanol and the drilling of a hole of approximately 0.1 cm at the edge of air sac of each selected viable egg. The amount of pSWCNT-Ag were determined by a previous research utilizing 62.5 µg/ml as the highest concentration to evaluate in vitro cytotoxicity on A549, J774, and Hep-2 cell lines [25]. A volume of 100 µl of PBS containing or 62.5 µg/ml pSWCNT-Ag was injected into the allantoic cavity of each egg using a tuberculin syringe. The hole was sealed with parafilm and eggs were immediately placed in the incubator. At day 20 of post-fertilization, chick embryos were taken from shells and humanely sacrificed to weigh and evaluate growth according to Hamburger and Hamilton standards [26]. The embryos were submitted to X-ray imaging using IVIS.

### Proteomic analysis

#### Protein extraction and 2D-gel electrophoresis

*Salmonella* Typhimurium bacteria were grown in the LB medium containing 0 or 25 µg/ml of pSWCNT-Ag. Cells were harvested at early stationary phase, washed three times with PBS, and frozen at − 80 °C until use. Total protein was extracted from frozen-thawed samples (Gene elute RNA/DNA/Protein purification plus kit; Sigma, MO, USA), precipitated (TCA/Acetone), and resulting pellets were dissolved in 220 µl rehydration sample buffer [7 M urea, 2 M thiourea, 4% CHAPS (w/v), and 20 mM dithiothreitol]. The isoelectric focusing (IEF) was carried out using IEF-strips (ReadyStrip IPG strips, 11 cm, pH 4–7, Bio-Rad; Hercules, CA, USA) and IEF conditions were 30 V for 12 h (rehydration), and 500 V for 15 min and 8000 V for 2.5 h with a total of 35 kVh. After IEF, the strips were then incubated for 15 min in equilibration buffer (6 M urea, 20% glycerol, 2% SDS, and 0.375 M Tris–HCL pH 6.8) containing 2% dithiothreitol (w/v), followed by additional incubation for 15 min in equilibration buffer containing 2.5% iodoacetamide (w/v). Electrophoresis was carried out by transferring the strips onto 4–20% gradient pre-casting gels (Criterion TGX Precast Gels 4–20%, Bio-Rad). Gels were then stained with Bio-safe Coomassie G-250 solution (Bio-Rad).

Three independent replicate gels per bacterial culture conditions (0 or 25 µg/ml pSWCNT-Ag) were obtained for scanning using Proteome Works Plus Spot Cutter (Bio-Rad). All images were aligned and protein spots were detected (PDQuest 2-D analysis software v7, Bio-Rad). Each spot was normalized based on the total valid spot intensity and protein quantitative analysis was performed by Student’s *t* test at *P *< 0.05. Significantly increased or decreased protein spots were extracted for protein identification using matrix-assisted laser desorption ionization time of flight/time of flight mass spectrometry (MALDI-TOF/TOF MS).

#### In-gel digestion, MALDI-TOF/TOF and functional analyses

In-gel tryptic digestion and subsequent MS spectra analysis were carried out at the Institute for Genomics, Biocomputing, and Biotechnology (IGBB), Mississippi State University. Briefly, the protein spots showing significant differences were automatically excised from the gels. Digested and dried peptides were dissolved in 2 μl of a saturated solution of α-Cyano-4-hydroxycinnamic acid in 50% acetonitrile and 0.1% of trifluoroacetic acid. As the internal standards, des-Arg-bradykinin (monoisotopic mass, 904.4681) and angiotensin I (1296.6853) were mixed with dissolved peptide samples which were loaded onto MALDI target plates.

Spotted peptide samples were analyzed by MALDI-TOF/TOF MS (4700 Proteomics Analyzer, Applied Biosystems, MA, USA). Monoisotopic peptide masses were selected in the mass range of 800–2500 Da with an acceleration voltage of 20 kV. The mass spectra were acquired by a cumulative average of 300 laser pulses, and protein identification was performed by peptide mass finger printing (MS-Fit program: http://prospector.ucsf.edu or MASCOT: http://www.matrixscience.com) with a mass tolerance of ± 50 ppm.

The classification of biological process and functional annotation of identified proteins were performed using the clusters of orthologous groups (COGs) analysis (https://www.ncbi.nlm.nih.gov/COG/). The subcellular localization was predicted using the pSORTb v3 (http://www.psort.org/psortb/), and protein interaction analysis was performed using the STRING v10.5 (https://string-db.org/).

### Statistical analysis

Statistical analyses were carried out using Graphpad Prism version 7.0.1. Significant differences were determined by Student’s *t* test with two-tailed nonparametric analysis (*P *< 0.05) and data were presented as mean ± standard deviation (SD).

## Results

### Characterization of pSWCNT-Ag and SWCNT-Ag

**Fig. 1:**
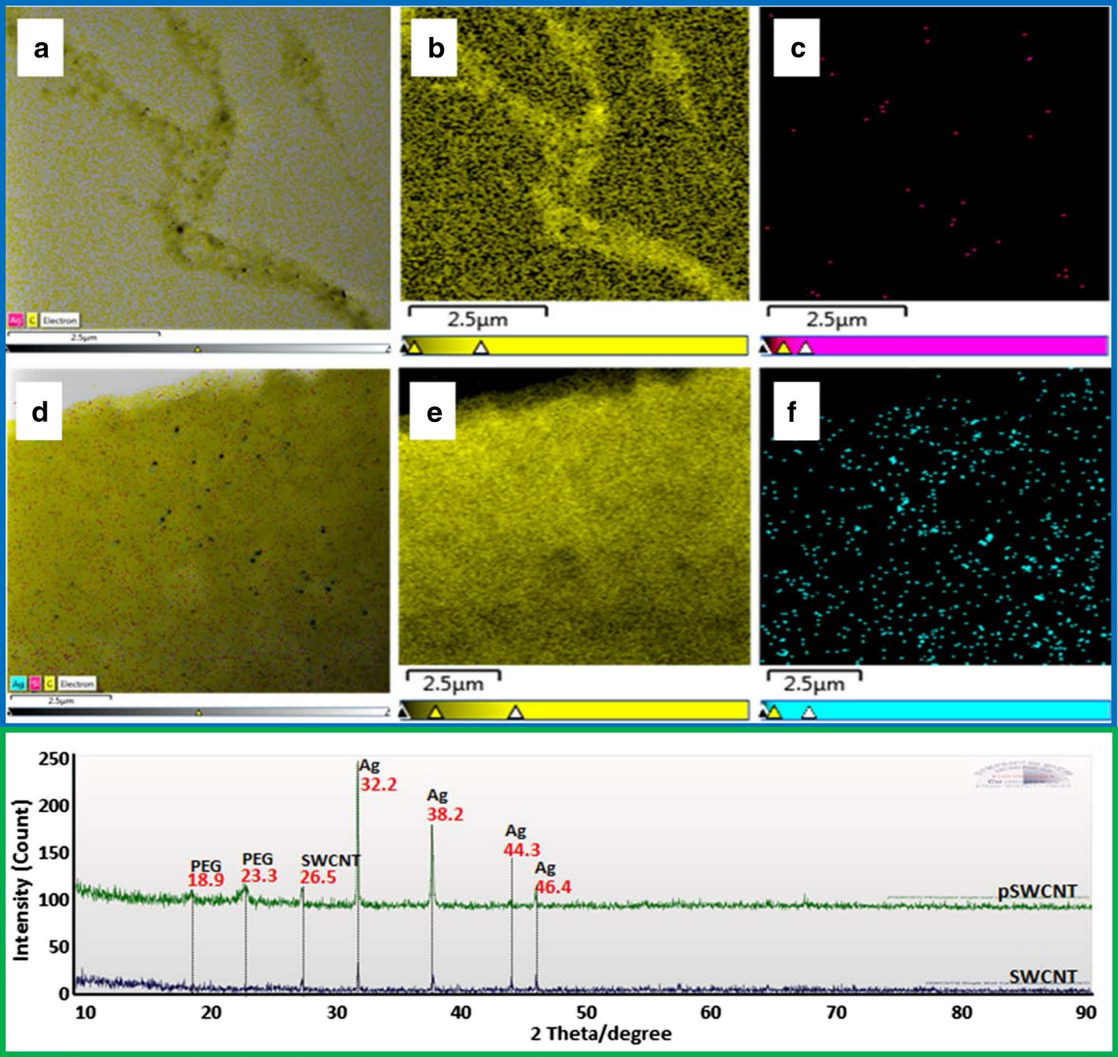
Energy dispersive X-ray spectroscopy (EDS) and X-ray diffraction (XRD) pattern analysis of pSWCNT-Ag and SWCNT-Ag. The upper panel (blue box) shows EDS mapping analysis of SWCNT-Ag (**a**–**c**) and pSWCNT-Ag (**d**–**f**). Micrographs **a**, **d** are merged images of carbon (**b**, **e**) and silver (**c**, **f**) atoms. The lower panel (green box) represents the XRD patterns depicting the presence of silver atoms (Ag) in both nanocomposites, and polyethylene glycol or PL-PEG 5000-amine (PEG) in pSWCNTs

Figure 1 (blue box) shows the EDS mapping images of the SWCNT-Ag (a–c) and pSWCNT-Ag (d–f) samples. The carbon ions representing the principal element of both nanocomposites are seen as yellow dots in micrographs b and e, while the deposition of silver ions are shown in micrographs c and f. Micrographs b/c and e/f are merged to form a and d, respectively. The XRD pattern analysis in Fig. 1 (green box) shows the presence of silver ions with four 2θ peaks at 32.2°, 38.2°, 44.3° and 46.4° in both nanocomposites, and an additional peak at 26.5° for SWCNT only. The successful pegylation is also observed through the detection of specific peaks (18.9° and 23.3°) in the pSWCNT-Ag alone, indicating the presence of the PL-PEG 5000-amine.

### Growth measurements of *E. coli*, *S*. Typhimurium and *S*. Anatum

**Fig. 2:**
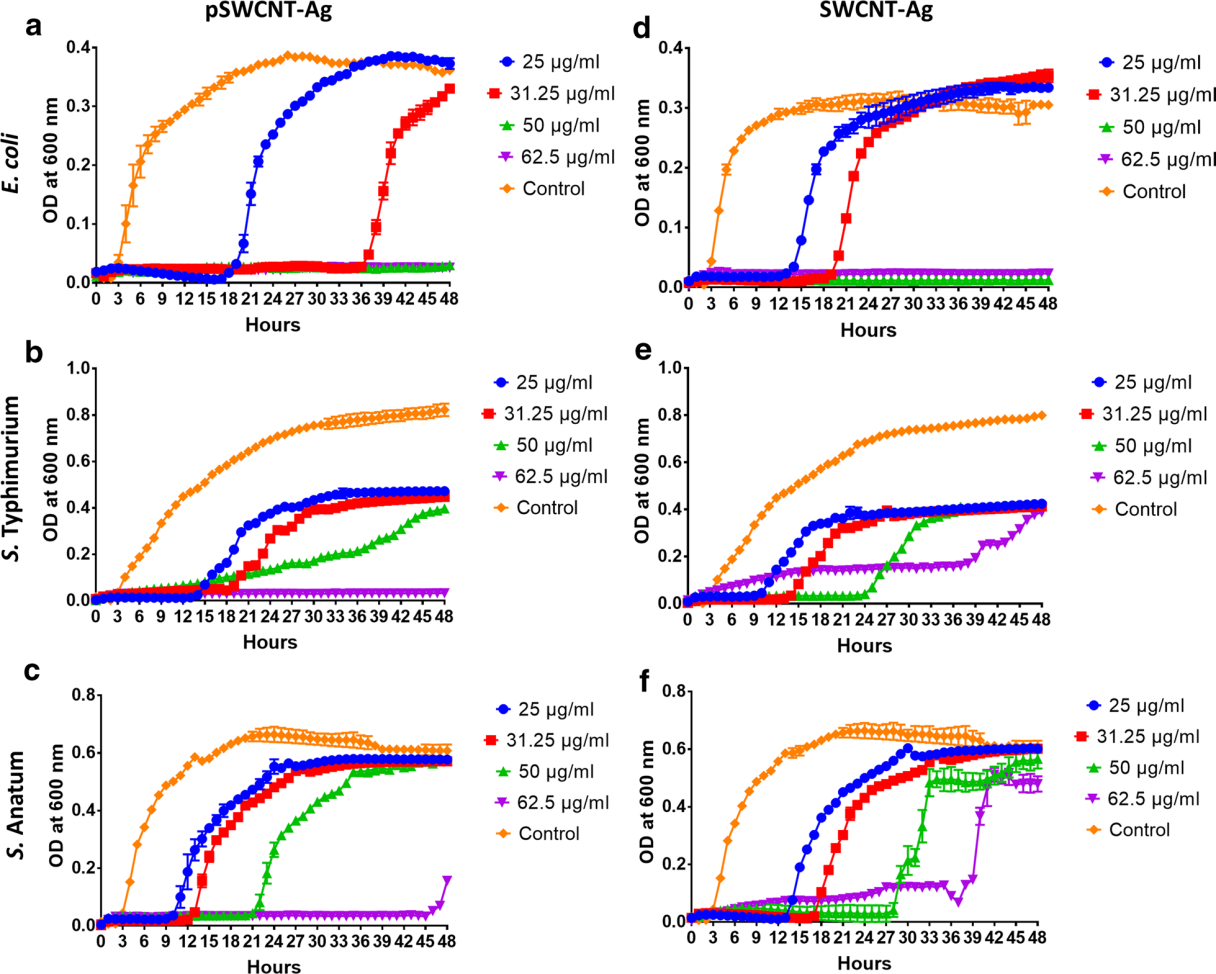
Effects of SWCNT-Ag and pSWCNT-Ag on bacterial growth. The graphs show optical density measurements (600 nm) of bacteria growth over 48-h exposure to various concentrations of pSWCNT-Ag (**a**–**c**) and SWCNT-Ag (**d**–**f**). Data are mean ± SD of three independent cultures of *E. coli* (**a**, **d**), *S.* Typhimurium (**b**, **e**) and *S.* Anatum (**c**, **f**)

The optical density results are summarized in Fig. 2. There were dose-dependent effects of pSWCNT-Ag and SWCNT-Ag on all bacteria growth, as referred to the significant delayed initiation of the exponential phases (or extended lag-phases) in comparison to their respective controls (Fig. 2). For instance, the exponential phase in *E. coli* started only after 18 and 36 h by the presence of 25 and 31.25 µg/ml pSWCNT-Ag, versus 3 h in the control. Similar patterns were observed with both Salmonellas, irrespective of the nanocomposite. The pSWCNT-Ag exhibited greater antibacterial potential than SWCNT-Ag, characterized by longer delayed exponential phase initiation (or longer lag-phases) in both *E. coli* and *S.* Typhimurium (Fig. 2). For example, *E. coli* in culture media containing 25 µg/ml pSWCNT-Ag had a delayed exponential initiation of 18 h (vs. 13 h for SWCNT), while the presence of 31.25 µg/ml provided 36 h (vs. 19 h) delayed. The *S.* Typhimurium growth curves clearly exemplify the greater effect of pSWCNT-Ag than that of SWCNT-Ag (delayed exponential phase initiation and complete growth inhibition at the highest pSWCNT-Ag). In addition, species-specificity responses of bacteria were observed at higher concentrations of either nanocomposite (50 and 62.5 µg/ml). The *E. coli* growth was completely inhibited by both concentrations of nanocomposites, while only 62.5 µg/ml pSWCNT-Ag provided similar effects on both Salmonellas. The concentration of 50 µg/ml pSWCNT-Ag and both 50 and 62.5 µg/ml SWCNT-Ag had limited to non-permanent inhibitory effects on *S.* Typhimurium and *S.* Anatum.

**Fig. 3:**
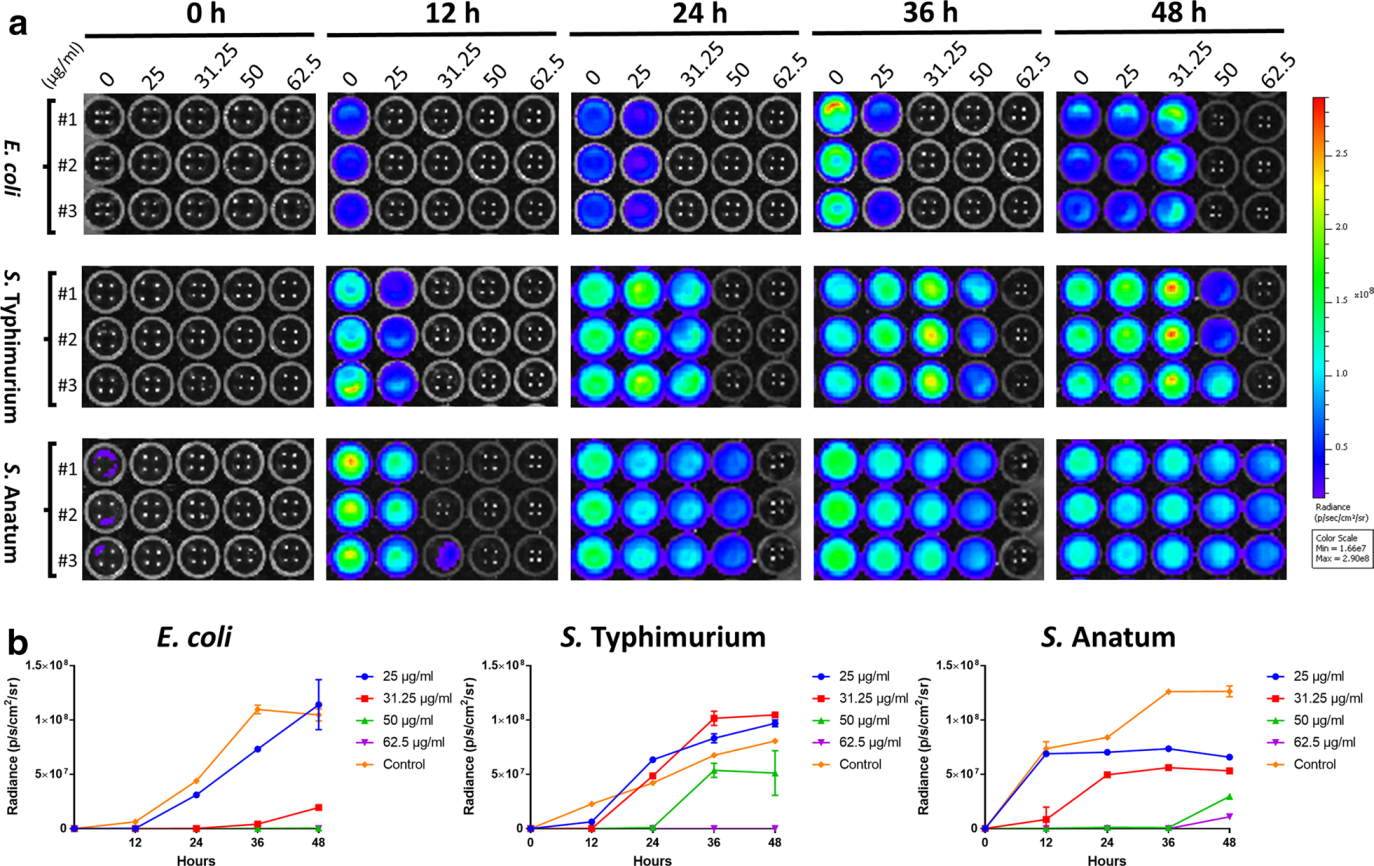
Real-time bioluminescence imaging of bacteria growth. Bioluminescence emission was imaged (**a**) and quantified (**b**) at 0, 12, 24, 36 and 48 h of culture. Bacteria (*E. coli*, *S.* Typhimurium and *S.* Anatum) exposed to SWCNT-Ag or pSWCNT-Ag showed similar patterns. Therefore, a representative culture plate containing bioluminescence-emitting bacteria under pSWCNT-Ag exposure is shown (**a**). Bioluminescence intensities (radiance) were plotted to generate the corresponding growth curves (**b**). Data (radiance) are mean ± SD of three independent cultures, with each bacteria strain plated in triplicate (#1, #2, and #3) per culture. The pseudo-color code scaling bar indicates the lowest (blue; 0.166 × 10^8^) to the highest (red; 2.9 × 10^8^) radiance

Bacteria exposure to both nanocomposites revealed similar bioluminescence imaging (BLI) patterns, and Fig. 3 is a culture plate with bacteria exposed to pSWCNT-Ag. The bioluminescence intensities of *E. coli*, *S.* Typhimurium and *S.* Anatum appeared in a dose-dependent manner, regardless of the nanocomposite but the pSWCNT-Ag showed most potent effects. Bacteria suspensions in higher pSWCNT concentrations had no bioluminescence emission above background (*E. coli* in 50 and 62.5 µg/ml, and *S.* Typhimurium in 62.5 µg/ml), while *S.* Anatum exhibited lower bioluminescence signals only under 62.5 µg/ml pSWCNTs.

**Fig. 4:**
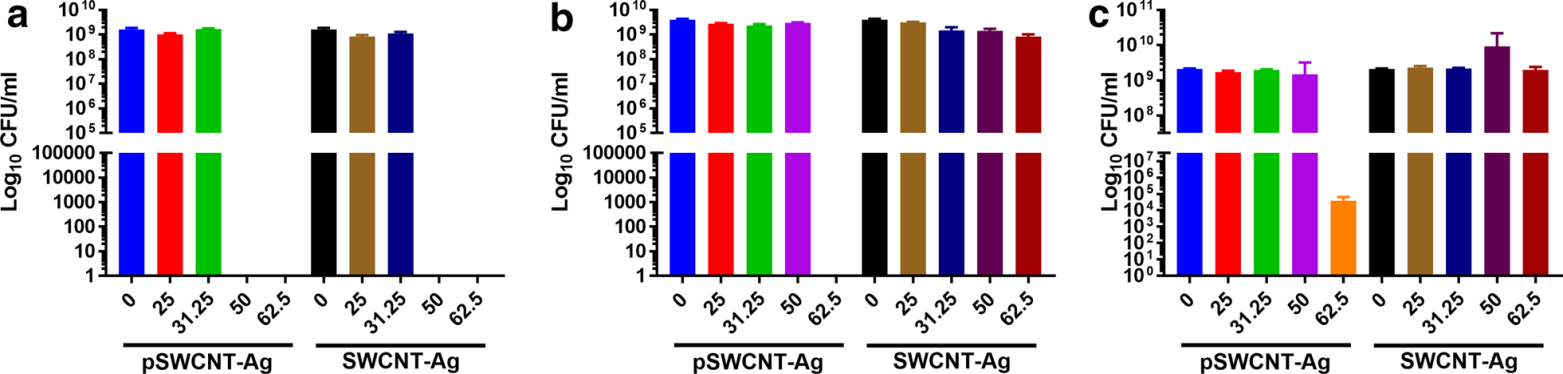
Bacterial survival after exposure to pSWCNT-Ag and SWCNT-Ag. Colony forming units (CFU/ml) were evaluated from each *E. coli* (**a**), *S.* Typhimurium (**b**), and *S.* Anatum (**c**) harvested after 48 h cultures in the presence of various concentration of pSWCNT-Ag and SWCNT-Ag (0, 25, 31.25, 50, and 62.5 µg/ml). Data are mean ± SD of three independent cultures

The CFU evaluations by the end of cultures (48 h) are summarized in Fig. 4. No CFU (living cells) were found after *E. coli* exposure to 50 or 62.5 µg/ml pSWCNT-Ag, or *S.* Typhimurium exposed to 62.5 µg/ml pSWCNT-Ag. However, CFU were counted in *S.* Anatum following exposure to 50 or 62.5 µg/ml pSWCNT-Ag. Additionally, bacteria exposed to lowest pSWCNT-Ag concentrations (≤ 50 µg/ml) had comparable CFU numbers with controls (P > 0.05).

### Evaluation of pSWCNT-Ag toxicity on developing chicken embryo

**Fig. 5:**
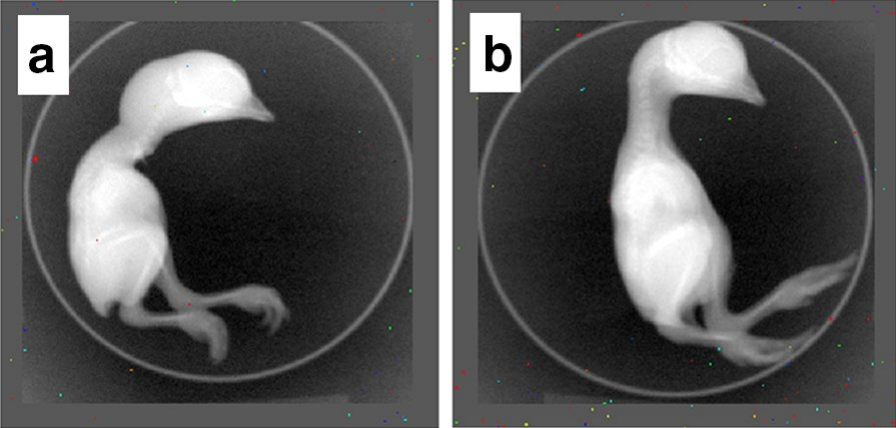
Toxicity evaluation of pSWCNT-Ag on developing chicken embryos. Fertilized chicken eggs were inoculated at 12th day of development with either PBS (Control) or pSWCNT-Ag within the allantoid. Eggs were broken at 20th day of development and fetuses were sacrificed for evaluation. Photographs are X-ray images (IVIS XRMS series III) of chicken controls (**a**) and exposed (**b**)

As seen in Fig. 5, *in ovo* injections of fertilized eggs with PBS or pSWCNT-Ag (62.5 µg/ml) on day 12 post-fertilization did not perturb embryo development. Both PBS (control) and pSWCNT-injected eggs were comparable, and chicks were alive at the collection on day 20. The weight of both control and pSWCNT-Ag chicks were not different (24.03 ± 1.57 and 23.13 ± 0.77 g, respectively; P > 0.05). Embryos of both groups exhibited similar skeletal structures and bone mass, with no structural abnormality observed through X-ray imaging (Fig. 5). The weight of various internal organs such as brain, hearts, liver, spleen and bursa of Fabricius harvested from both control and treated chicks showed no significant differences (P > 0.05; data not shown).

### Proteomic analyses and identification of differentially expressed proteins

**Fig. 6:**
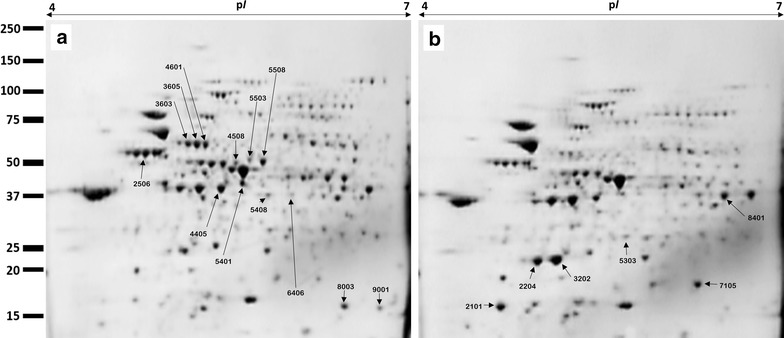
Representative two-dimensional electrophoresis gels of control (**a**) and pSWCNT-Ag-exposed (**b**) *S*. Typhimurium. Down- and up-regulated protein spots are shown in (**a**) and (**b**), respectively

**Fig. 7:**
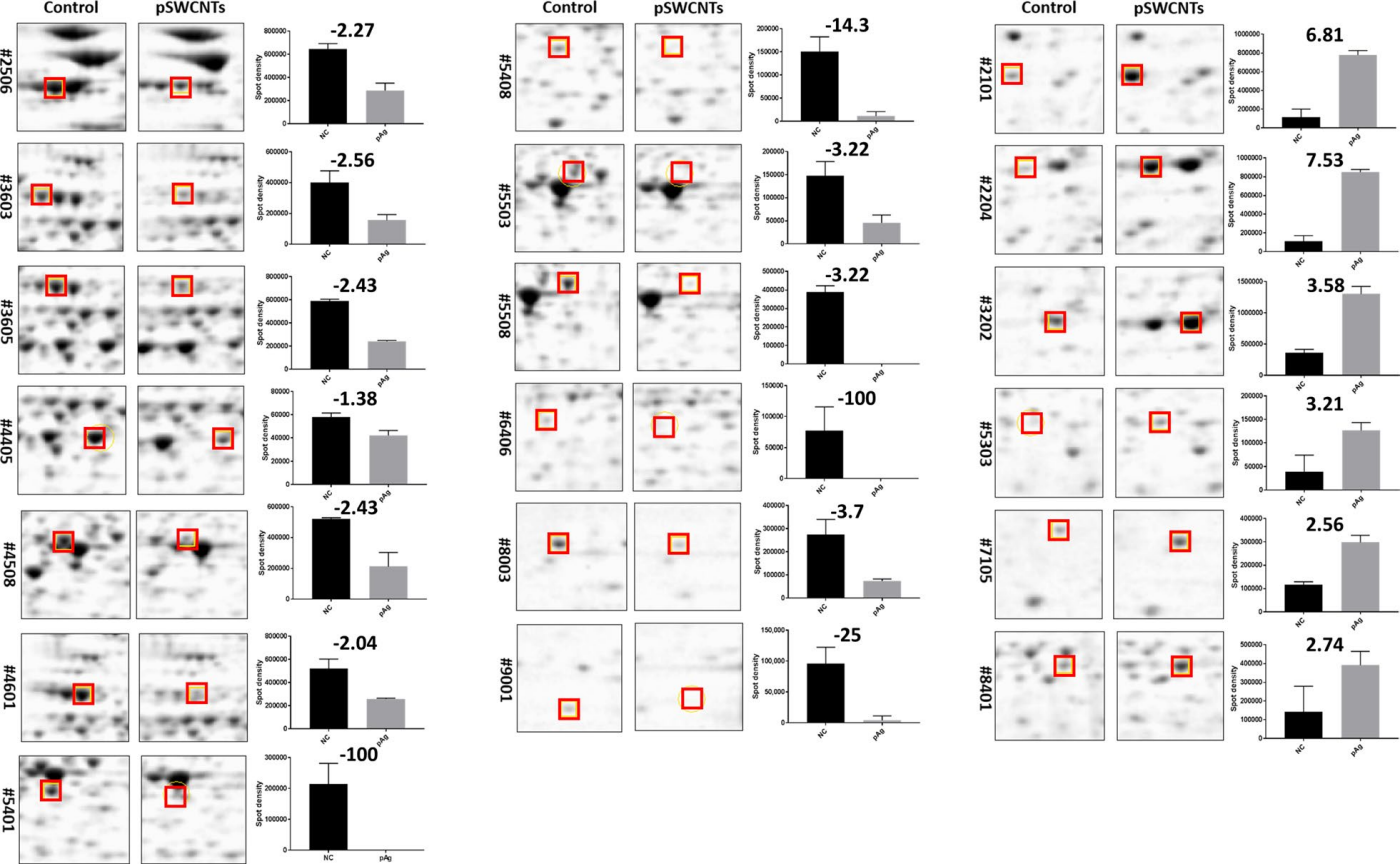
Comparative analysis of differentially detected protein spot intensities. A total of 19 representative altered or spiked protein spots are shown with negative or positive fold change values, respectively. Quantification of spot intensities were analyzed by PDQuest 2-D software and ratios of spot intensity (exposed/control) are shown in each graph. Data are mean ± SD of three gels derived from three independent bacteria cultures. Differential regulation was called with *P *< 0.05 (Student’s *t* test)

Figure 6 shows representative gel electrophoreses of control or treated *S.* Typhimurium with 25 µg/ml pSWCNT-Ag. The matching of three independent and highly reproducible gel replicates per treatment group was performed, followed by quantitative spot intensity analyses that revealed a total of 114 protein spots being significantly differentially expressed between control and treated samples (*P *< 0.05). Among them, 12 spots (2506, 3603, 3605, 4405, 4508, 4601, 5401, 5408, 5503, 6406, 8003, and 9001) were down-regulated (Fig. 6a), with ten identified as flagellin FliC, aspartate ammonia-lyase, outer membrane protein A, adenylosuccinate synthetase, arginine deiminase, ornithine carbamoyltransferase, carbamate kinase, l-asparaginase 2, universal stress protein F, and ethanolamine utilization protein EutM. In contrast, 6 spots (2101, 2204, 3202, 5303, 7105, and 8401) were up-regulated (Fig. 6b) and five were identified as outer membrane protease, alkyl hydroperoxide reductase subunit C, propanediol utilization microcompartment protein PtuB, DNA starvation/stationary phase protection protein, and aldehyde dehydrogenase. Quantitative analyses of protein spots revealed downregulation of 1.4- to 100-fold and upregulation of 2.56- to 7.53-fold changes (Fig. 7).

Table 1 summarizes the characteristics of identified proteins. All proteins showed 100% protein score confidence interval (CI) % and total ion CI %. Functional analyses of identified proteins showed association with different sub-cellular localization such as cytoplasm (53%), outer membrane (13%), extracellular (7%), periplasm (7%) and unknown (20%). Functional prediction using COGs analysis indicated that proteins were involved in amino acid transport and metabolism (E, 40%), cell wall/membrane/envelope biogenesis (M, 13%), translation, ribosomal structure and biogenesis (J, 13%), cell motility (N, 7%), nucleotide transport and metabolism (F, 7%), defense mechanisms (V, 7%), secondary metabolites biosynthesis, transport and catabolism (Q, 7%) and energy production and conversion (C, 7%).

**Table 1:** Differentially expressed proteins in *S.* Typhimurium exposed to pSWCNT-Ag

Spot ID	Protein name	NCBI no.	Abbr.	Func.^a^	Local.^b^	MW (kDa)^c^	p*I*^d^	No. of peptide matched	Protein score^e^	Total ion score^f^	Protein score CI %^g^	Total ion CI %^h^
Down-regulated												
2506	Flagellin FliC	gi|446001950	hag	N	Extracellular	51.5	4.79	16	930	830	100	100
3603 3605 4601	Aspartate ammonia-lyase	gi|445991585	aspA	E	Cytoplasmic	52.2	5.15	17	450	335	100	100
4405	Outer membrane protein A	gi|487406894	ompA	M	Outer membrane	37.4	5.6	16	571	449	100	100
4508	Adenylosuccinate synthetase	gi|446450117	purA	F	Cytoplasmic	47.3	5.31	21	265	116	100	100
5503 5508	arginine deiminase	gi|446332991	STM4467	E	Cytoplasmic	45.5	5.47	22	774	586	100	100
5401	Ornithine carbamoyltransferase	gi|446159176	STM4465	E	Cytoplasmic	36.7	5.28	11	585	514	100	100
5408	Carbamate kinase	gi|446350896	STM4466	E	Cytoplasmic	33.3	5.45	13	399	307	100	100
6406	l-Asparaginase 2	gi|446316334	ansB	E	Periplasmic	36.9	5.84	13	347	262	100	100
8003	Universal stress protein F	gi|447005040	uspF	J	Unknown	15.7	5.93	11	406	296	100	100
9001	Ethanolamine utilization protein EutM	gi|446309861	eutM	E	Unknown	9.8	6.06	6	164	110	100	100
Up-regulated												
2101	Outer membrane protease	gi|446639417	ompX	M	Outer membrane	18.4	5.74	9	500	425	100	100
2204 3202	Alkyl hydroperoxide reductase subunit C	gi|445974947	ahpC	V	Cytoplasmic	20.7	5.03	10	437	354	100	100
5303	Propanediol utilization microcompartment protein PduB	gi|446019642	pduB	Q	Unknown	27.9	5.21	9	378	318	100	100
7105	DNA starvation/stationary phase protection protein	gi|446022950	dps	J	Cytoplasmic	18.7	5.73	14	476	336	100	100
8401	Aldehyde dehydrogenase	gi|446075650	gapA	C	Cytoplasmic	35.5	6.33	20	572	393	100	100

^a^Functional classification was performed using COGs (Clusters of Orthologous Groups) functional annotation. *C* energy production and conversion, *E* amino acid transport and metabolism, *F* nucleotide transport and metabolism, *J* translation, ribosomal structure and biogenesis, *M* cell wall/membrane/envelope biogenesis, *N* cell motility, *Q* secondary metabolites biosynthesis, transport and catabolism, *V* defense mechanisms

^b^Subcellular localization was predicted using pSORTb version 3.0

^c^Predicted molecular weight (MW) and ^d^ isoelectric point (p*I*)

^e^Protein scores are derived from ion scores as a non-probabilistic basis for the ranking protein hits. Ions score is − 10 log(*P*), where *P* is the probability that the observed peptide match is a random event (http://www.matrixscience.com/help/interpretation_help.html)

^f^Total ion score is calculated by weighting ion scores for all individual peptides matched to the protein that is associated with this peptide and MS/MS spectrum (http://www.matrixscience.com/help/interpretation_help.html)

^g^Confidence interval % (CI %) rates the confidence level of the protein score [MS] or ion score [MS/MS]. The CI % is the statistical calculation based on the distribution of the probability that enables to compare the searched data base with the number of submitted mass spectra for the database searches

**Fig. 8:**
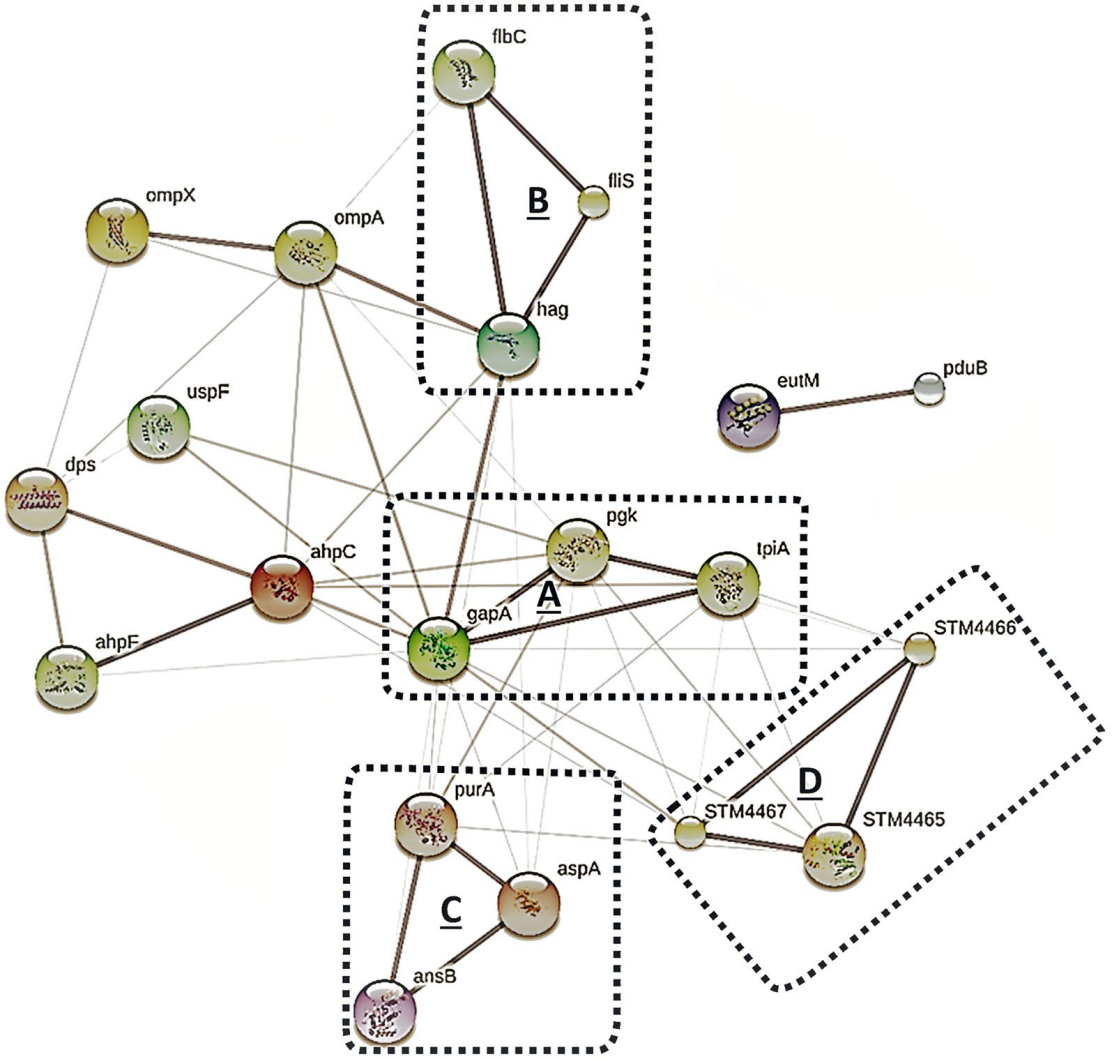
Interaction network analysis of differentially regulated proteins under pSWCNT-Ag. Four representative networks (**a**–**d** *P *= 0.0198) were predicted using STRING software (http://string-db.org). **a** glycolysis/gluconeogenesis, **b** Flagellar assembly pathway, **c** alanine, aspartate and glutamate metabolism, and **d** Arginine and proline metabolism

The KEGG pathway analysis revealed high relationships between up- or down-regulated proteins, with four major pathways identified (P < 0.05; Fig. 8). The glycolysis/gluconeogenesis (A: gapA, pgk, and tpiA) pathway was increased, while the flagella assembly (B: flbC, fliS and hag), alanine, aspartate and glutamate metabolism (C: ansB, aspA, and purA), arginine and proline metabolism (D: STM4465, STM4466, and STM4467) pathways were decreased after *S.* Typhimurium culture in the presence of pSWCNT-Ag.

## Discussion

Numerous studies have reported single-walled carbon nanotubes (SWCNTs) and silver nanoparticles (AgNP) as excellent candidates for alternative therapeutic agents to antibiotics [14, 15, 18]. Additionally, the formation of hydrophilic and functionalized nanocomposites such as pSWCNT-Ag and PLGA-SWCNT (poly-lactic-co-glycolic acid) can enhance antimicrobial strengths as compared to single unique nanoparticles [14, 27]. In the present study, the presence of SWCNT-Ag or pSWCNT-Ag dose-dependently inhibited growth kinetics of tested foodborne bacteria (*E. coli*, *S.* Typhimurium and *S.* Anatum), as observed through optical density (at 600 nm) and CFU measurements, both standard approaches to evaluate bacterial growth. However, the dose-dependent increased darkness of culture media together with the nanocomposite concentrations interfered with OD analyses, making bioluminescence imaging an excellent approach to precisely evaluate bacteria growth due to intrinsic real-time expression of the lux gene. Consequently, bioluminescence imaging allowed for early detection of bacteria growth than the OD method, regardless of the turbidity of the culture medium.

In this study, all analytical approaches confirmed the greater bactericidal effects of pSWCNT-Ag than SWCNT-Ag, which could be attributed to reduced agglomerates, higher dispersed and available pSWCNT-Ag to target bacteria [25, 28]. The observed antibacterial effects on *E. coli* and *S.* Typhimurium were in agreement with previous studies [25, 29], and the growth patterns differed across bacteria strains, depending on the pSWCNT-Ag concentrations. Despite *S.* Anatum showing the highest resistance against both nanocomposites, duration of the lag phases or initiation of the exponential phases of all bacteria strains were significantly and dose-dependently increased, in comparison to their respective controls. In all bacteria strains, pSWCNT-Ag consistently maintained the stronger effects than SWCNT-Ag, suggesting the possibility for decreased antibiotic concentrations when combining them pSWCNT-Ag nanocomposites for disease control or disinfection process. Yet, there are conflicting reports regarding the antibacterial effects of the silver nanomaterial itself that may affect the efficacy of derived carbon nanocomposites. These discrepancies are attributable to bacteria species and/or nanomaterial physico-chemistry characteristics such as surface/volume, shape, size and zeta potential that may vary among studies [30–32]. The recent studies suggested 50 and 62.5 µg/ml as the most effective inhibitory concentrations for all tested bacteria strains, not affecting eukaryotic cells, such as human cell lines [25, 33, 34],

In the current study, the potential toxic effects of pSWCNT-Ag nanocomposites were evaluated on developing chicken embryos, reported as an outstanding animal model to assess the toxicity of organic and inorganic compounds [35], due to their ability to overcome the blood–brain barrier and cause developmental toxicity during embryogenesis [36]. Here, the *in ovo* injection of pSWCNT-Ag at 62.5 µg/ml (the highest concentration in bacterial evaluations) did not affect embryo development prior to hatching. Chicks harvested from control and injected eggs were still alive and comparable based upon the developmental stage 45 of the Hamburger and Hamilton standards, referring to eggs with half of the yolk sac enclosed in the body cavity and chorio-allantoic membrane glued in embryo with less blood [26]. There were no differences in terms of bone mass observation through X-ray imaging, embryo weight, embryo size and weight of various internal organs of both control and injected chicken. Most importantly, our results reveal the non-toxicity of pSWCNT-Ag to during *in ovo* embryo-fetal development.

The selective targeting of prokaryote cells implies specific mechanisms of action of pSWCNT-Ag nanocomposites. Carbon-based nanotubes are known to induce growth inhibition through direct contact with bacteria, leading to various cellular perturbations and cell death [14]. A genomic DNA microarray study has revealed the downregulation of a large number of genes encoding flagella components of *Bacillus cereus* after exposure to silver nanoparticles [37]. A recent study has reported the inhibition of mRNA expression levels of genes involved in the regulation of normal physiology (*ybeF*), quorum sensing (*sdiA*), outer membrane structure (*safC*), invasion (*ychP*) and virulence (*safC*, *ychP*, *sseA* and *sseG*) of S. Typhimurium exposed to pSWCNT-Ag [25]. These later findings prompted us to use the foodborne pathogenic and multidrug resistant S. Typhimurium as a bacterium model to investigate the protein changes. As the functional unit of the cell, the study of the bacteria global proteomic analysis provides a unique and important knowledge of the molecular mechanisms of having direct effects on *S.* Typhimurium, as observed in this study. A total of 15 proteins playing crucial roles in bacterial growth, survival, and division were differentially regulated by the presence of pSWCNT-Ag.

These proteins were predicted to significantly alter the glycolysis/gluconeogenesis, alanine, aspartate and glutamate metabolism, arginine and proline metabolism, and flagella assembly. Previous studies have demonstrated the adverse effect of l-asparaginase 2 (AnsB) and aspartate ammonia-lyase (AspA) down-regulation in *S.* Typhimurium. In support of our findings, the deletion of l-*asparaginase 2* (AnsB) enables bacteria to avoid recognition and clearance by the host immune system [38], while lower aspartate ammonia-lyase (aspA) levels in cells reduce communications between neighboring bacteria, due to the conversion of aspartate into fumarate with decreased production of ammonia used as quorum sensing molecule in bacteria [39, 40]. In the same line, the downregulation of adenylosuccinate synthetase (purA), having a crucial role in the purine biosynthesis, reduces bacterial growth and attenuates pathogenicity at all stages of infection such as colonization, invasion, and internalization [41, 42]. Likewise, the down-regulation of fermentative enzymes appears in favor of bacterial growth inhibition observed under the pSWCNT-Ag exposure. These enzymes corresponded to arginine deiminase (STM4467), ornithine carbamoyl-transferase (STM4465) and carbamate kinase (STM4466), participating into arginine and proline metabolism, as well as the arginine deiminase (ADI or STM4467) system that is important for bacterial protection to acidic extracellular damage [43]. Indeed, the ADI system converts l-arginine into ornithine, ammonia, carbon dioxide, and energy (ATP), all products contributing to acidic pH neutralization within the phagosome and therefore the survival of *S.* Typhimurium in the host cell (i.e., macrophage) [44, 45]. Other down-regulated proteins corresponded to flagellin FliC (hag, spot2506, 2.3×), outer membrane protein A (ompA, spot4405, 1.4×), universal stress protein F (uspF, spot8803, 1.4×), ethanolamine utilization protein EutM (eutM, spot9001, 25×), and outer membrane protein A (ompA, spot4405, 1.38×). These proteins are involved in various bacterial function such as locomotion (flagellin FliC—[37]), bacteria growth and colonization (eutM—[46]), and survival in hostile environments such as pH, osmolality and temperature (protein F—[47]), bacterial structure, survival and communications in hostile environments, and nutrient transportation (ompA—[48, 49]). Altogether, these results support the antibacterial effects of pSWCNT-Ag through the suppression of various metabolic pathway regulations, cell motility, and biofilm formation, resulting in the attenuation of virulence and reduction of bacterial growth. In support of these predictions, both ultrastructure (scanning electron microscopy) and nanoscale (atomic force microscope) imaging have shown silver nanoparticle attachments from the basal body to the distal end of the flagellum of bacteria, leading to serious damages and reduced motility [50, 51].

As a compensatory bacterial response to pSWCNT-Ag exposure, upregulations of outer membrane protease (ompX, spot2101, 6.81×), alkyl hydroperoxide reductase subunit C (ahpC, spot2204, 7.53×), propanediol utilization microcompartment protein pduB (pduB, spot5303, 3.21×), DNA starvation/stationary phase protection protein (dps, spot7105, 2.56×) and aldehyde dehydrogenase (gapA, spot8401, 2.74×) were observed. These increases help to protect bacteria through membrane rearrangements by removal or destruction of damaged proteins (ompX—[52]), elimination of reactive oxygen species that can be induced by silver nanomaterials (ahpC—[53, 54]), optimization of metabolic pathways (pduB—[55]), DNA protection from oxidative damages and survival to starvation (dps—[56]), improved energetic metabolism (gapA—[57]). Overall, it appears that under pSWCNT-Ag exposure, *S.* Typhimurium develops resistance through protection against oxidative respiratory stress and supplementary gain of energy and nutrients via other sources such as nitrogen, carbon and energy from ethanol, 1-propanol and 2-propanol.

## Conclusions

The present study confirms the dispersed SWCNT-Ag (or pSWCNT-Ag) has an excellent antimicrobial property against foodborne pathogens with undetectable toxic effects during *in ovo* embryo-fetal development of chicken. Most importantly, the study provides, in our knowledge, the first and comprehensive proteomic analysis revealing significant protein changes and predicted biological functions in *S.* Typhimurium bacteria following exposure to pSWCNT-Ag. With all tested foodborne bacteria showing extended lag phases or initiation of exponential growth phases and lack of eukaryotic cell toxicity, the current study contributes to support the use of dispersed nanocomposites, such as the pSWCNT-Ag, for disinfection and therapeutic procedures.

## Abbreviations

Ag: silver
COG: clusters of orthologous groups
ATCC: American type culture collection
*E. coli*: *Escherichia coli*
CFU: colony forming unit
FT-IR: Fourier transform infrared spectroscopy
IVIS: in vivo imaging system
LB: Luria–Bertani broth
MALDI-TOF/TOF MS: matrix assisted laser desorption/ionization time of flight/time of flight mass spectrometry
PBS: phosphate-buffered saline
*S. Typhimurium*: *Salmonella enterica* serovar Typhimurium
SWCNT: single walled-carbon nanotube
SWCNT-Ag: silver coated single walled-carbon nanotube
pSWCNT-Ag: pegylated (polyethylene glycol) silver coated single-walled carbon nanotube
PL-PEG: phospholipid-poly ethylene glycol-amine
TEM: transmission electron microscopy
EDS: energy dispersive X-ray spectroscopy
XRD: X-ray diffraction
